# Prevalence of Missing Values and Protest Zeros in Contingent Valuation in Dental Medicine

**DOI:** 10.3390/ijerph18147219

**Published:** 2021-07-06

**Authors:** Pedram Sendi, Arta Ramadani, Michael M. Bornstein

**Affiliations:** 1 Department of Oral Health and Medicine, University Center for Dental Medicine Basel UZB, University of Basel, Mattenstrasse 40, 4058 Basel, Switzerland; arta.ramadani91@gmail.com (A.R.); michael.bornstein@unibas.ch (M.M.B.); 2 Institute for Clinical Epidemiology, Basel University Hospital, Spitalstrasse 12, 4031 Basel, Switzerland

**Keywords:** dental medicine, contingent valuation, willingness-to-pay, health services research, oral public health, missing values, protest zeros, economic evaluation, health state utility

## Abstract

Background: The number of contingent valuation (CV) studies in dental medicine using willingness-to-pay (WTP) methodology has substantially increased in recent years. Missing values due to absent information (i.e., missingness) or false information (i.e., protest zeros) are a common problem in WTP studies. The objective of this study is to evaluate the prevalence of missing values in CV studies in dental medicine, to assess how these have been dealt with, and to suggest recommendations for future research. Methods: We systematically searched electronic databases (MEDLINE, Web of Science, Cochrane Library, PROSPERO) on 8 June 2021, and hand-searched references of selected reviews. CV studies in clinical dentistry using WTP for valuing a good or service were included. Results: We included 49 WTP studies in our review. Out of these, 19 (38.8%) reported missing values due to absent information, and 28 (57.1%) reported zero values (i.e., WTP valued at zero). Zero values were further classified into true zeros (i.e., representing the underlying preference of the respondent) or protest zeros (i.e., false information as a protest behavior) in only 9 studies. Most studies used a complete case analysis to address missingness while only one study used multiple imputation. Conclusions: There is uncertainty in the dental literature on how to address missing values and zero values in CV studies. Zero values need to be classified as true zeros versus protest zeros with follow-up questions after the WTP elicitation procedure, and then need to be handled differently. Advanced statistical methods are available to address both missing values due to missingness and due to protest zeros but these are currently underused in dental medicine. Failing to appropriately address missing values in CV studies may lead to biased WTP estimates of dental interventions.

## 1. Introduction

In times of increasing pressures to contain healthcare resource consumption, there is an increasing interest in valuing dental healthcare goods and services using economic methods such as contingent valuation (CV) [1,2]. CV studies assess the value of a commodity by asking the respondent the maximum in monetary terms he is willing to pay (WTP) to receive a good or service, or alternatively, the minimum he is willing to accept (WTA) to forgo the good or service offered [3,4]. CV studies were first used in environmental economics for valuing goods and services for which no free real-world market exists, but have then gained more attention in healthcare, and more recently, also in dental medicine [1,2,5,6,7]. Other economic methods for valuing health states and interventions such as the time-trade-off method or standard-gamble method allow one to estimate health state utilities that are often used to compute composite outcomes such as quality-adjusted life-years (QALYs), or more oral health-related outcomes such as quality-adjusted tooth-years (QATYs) or quality-adjusted prosthesis-years (QAPYs) [8,9,10]. However, these outcomes do not allow a direct comparison of the benefit of a healthcare good or service with its associated costs. CV studies, on the other hand, ask the respondent to explicitly state their preference in monetary terms, and can therefore be used in cost-benefit analyses where the costs are directly compared with the benefits in monetary terms [11,12].

A common problem in many studies is missing values, usually because of absent information [13,14,15]. In CV studies, this may be due to the respondent not answering the WTP question because they may not have understood the question or because he or she is reluctant to answer the question. Pilot testing a WTP instrument may, therefore, prove to be helpful in identifying possible problems in the WTP elicitation procedure. Missing values are classified as missing completely at random (MCAR), missing at random (MAR), or missing not at random (MNAR) [13,14]. MCAR implies that the reason for missingness is unrelated to the data and caused by chance alone, i.e., the respondents with absent information do not differ from those who answered the WTP question. In that case, a complete case analysis is often performed, leaving out the subset of respondents with missing data [16]. Besides the assumption of MCAR being often unrealistic in practice, a complete case analysis leads to a loss of power [13,14,16]. MAR implies that the probability of missingness can be explained by the observed data. If, for example, younger respondents produce less missing values in WTP elicitation than older respondents, and MCAR within each age group is assumed, then we can use age group and statistical missing data methods, such as multiple imputation (MI), to replace missing WTP data with reasonable values [13,17,18]. MNAR implies that the reason for missingness cannot be explained by the observed data. MNAR is more complex and, in that case, more research is needed to elaborate on the reasons for missingness; usually sensitivity analyses need to be performed when missing data methods are used. However, when many variables with respect to patient characteristics are recorded in a study, usually MAR is assumed and methods such as MI are used to generate a reasonable dataset for further analysis [13,14,17].

A further problem specific to CV studies are zero values [7,19,20]. Respondents may value a good or service with zero WTP, implying that they are not willing to pay any amount of money for the good or service provided. This may be due to economic reasons, i.e., because the commodity has no value to them (i.e., true zeros), or due to non-economic reasons. Non-economic reasons may, for example, represent their opinion that the government or insurance should pay for the service [19,20]. Their zero WTP, therefore, does not reflect their true underlying valuation of the commodity, and is hence termed as a protest zero or protest answer. Protest zeros can be interpreted as missing values due to false information. Although several statistical methods such as the Heckman model have been suggested to handle zero values in CV studies, more recently, it has been proposed to replace protest zeros using missing data methods such as MI, assuming MAR, to generate a reasonable dataset for further analysis [19]. Since the literature in CV studies in dental medicine has been rapidly increasing in recent years, we believe that a review of the methods used to address missing data in the hitherto published studies would help to shed light on the current status quo.

The aim of the present study is, therefore, to (i) estimate the prevalence of missing and zero values in CV studies in dentistry, (ii) evaluate whether a distinction was made between true zeros and protest zeros in CV, (iii) evaluate how missing and zero values were dealt with in CV, and (iv) suggest recommendations for future research.

## 2. Materials and Methods

### 2.1. Systematic Literature Search

We searched the databases MEDLINE via PubMed and Web of Science on 8 June 2021 using a combination of Medical Subject Headings (MeSH in PubMed) and general search terms (PubMed and Web of Science). The search strategy for Web of Science included the query: (“Willingness-to-pay” OR WTP OR “Willingness to accept” OR WTA OR “Contingent valuation” OR “Conjoint analysis” OR “Cost benefit analysis” OR “Discrete choice experiment” OR “Monetary value”) AND Topic: (“dent*). The search strategy for PubMed included the query: (“Willingness-to-pay” OR WTP OR “Willingness to accept” OR WTA OR “Contingent valuation” OR “Conjoint analysis” OR “Cost benefit analysis” OR “Discrete choice experiment” OR “Monetary value”) AND (Dentistry (MeSH) or “dent”). In addition, we hand-searched the references of two published systematic reviews on WTP estimates for dental interventions [1,2]. We also searched the Cochrane Library (www.cochranelibrary.com, accessed 8 June 2021) and the PROSPERO database (www.crd.york.ac.uk/prospero, accessed 8 June 2021) for published or eventual ongoing studies that assessed WTP using CV as a stated preference method on 8 June 2021. We did not limit the time span for search in the respective databases.

After exclusion of duplicates, titles and abstracts were screened by two reviewers (A.R. and P.S.) and further considered using the following eligibility criteria: publication in English (considered as the scientific world language); empirical studies evaluating a clinical intervention or a dental health state; reporting WTP estimates using CV methodology for a dental service or good, and full-text available. If, from the abstract and titles eligibility was not clear, the full-text article was screened to assess eligibility. For each included article, we extracted the following study characteristics: first author, year of publication, country, dental good or service provided, sample size, CV elicitation method, reporting of missing values, how missing values were dealt with, reporting of zero values, whether a distinction was made between true zeros and protest zeros, and statistical methodology used. In order to minimize extraction errors, all data were extracted by two independent reviewers twice and then compared. Differences were resolved by discussion.

### 2.2. Analysis of Extracted Data

The proportion of missing values due to missingness (i.e., absent information) and zero values were collected from the included CV studies, and 95% confidence intervals were estimated for each statistic. In addition, the proportion of studies that made a distinction between true zeros and protest zeros (i.e., missing values due to false information) and the respective 95% confidence intervals were estimated. The statistical analyses were performed using R and RStudio (Version 1.2.1335, RStudio, Inc., Boston, MA, USA).

## 3. Results

Databases were searched on 8 June 2021 (MEDLINE, Web of Science, Cochrane Library and PROSPERO database). A total of 1705 records were initially identified through database searches (see Supplementary Material), with 1494 remaining after removal of duplicates for title and abstract screening. A total of 65 articles were included for full-text screening from database searches and 15 articles from citation searches, and of these, 49 remained for qualitative and quantitative analysis (see Figure S1 in Supplementary Material). Figure S1 displays the PRISMA 2020 (Preferred Reporting Items for Systematic Reviews and Meta-Analyses) flow diagram.

Table 1 displays the descriptive characteristics of the studies included in this study. Missing values due to absent information (i.e., missingness) was reported in 19 out of 49 studies, corresponding to a prevalence of 38.8% (95% CI: 25.5–53.8%). Zero values in WTP estimates were reported in 28 out of 49 studies (Table 1), resulting in a prevalence of 57.1% (95% CI: 42.3–70.9%). Out of the 28 studies that reported zero WTP values, only 9 studies made a distinction between true zeros and protest zeros (32.1%, 95% CI: 16.6–52.4%) (Table 1). Only one study used MI to replace missing values due to missingness, whereas in the remainder of the studies, a complete case analysis was performed (Table 1). When zero values were included in the statistical analysis, one study used a Heckman selection model, whereas six studies applied a Tobit regression model to analyze the data (Table 1). 

## 4. Discussion

In the present study, it was shown that a substantial proportion of CV studies (38.8%) reported missing values due to absent information, and even a larger proportion (57.1%) reported zero values. Zero values were further classified into true zeros (i.e., representing the underlying preference of the respondent) or protest zeros (i.e., false information as a protest behavior) only in less than one-third of the studies reporting zero values. Most studies used a complete case analysis to address missingness while only one study used MI [44].

### 4.1. Missing Values

Missing values are a common problem in medical and dental research [15,70], and they occur when information on a specific variable is absent and not recorded in the dataset. There may be different reasons for missing values in CV studies: (i) the respondent refuses to respond to the question, (ii) the respondent does not understand the WTP exercise and therefore does not provide a WTP value, or (iii) data may get lost due to investigator or computational error. If data are MCAR, then the dataset without missing values is a representative subsample of the complete dataset, and missingness does not depend on the observed as well as unobserved data [13,70]. If data are MAR, the probability of missing depends on the observed data, i.e., missing data can be explained by the observed variables and are independent of unobserved variables [13,14]. This is usually the most common assumption about the underlying mechanism of missing data. If missing data are neither MCAR or MAR, then they are MNAR [13]. MNAR implies that the probability of missing data cannot be explained by observed data and that the probability of missingness depends on the value of the missing data itself [13,14,15,70].

There are different approaches to handle missing data. The most commonly used method is complete case analysis where incomplete records are excluded from the analysis [16]. This approach, however, assumes MCAR and leads to a loss of power. Another approach is simple imputation such as mean value imputation [13]. Hereby the missing value is replaced with the mean value of those records where information is available. This approach, however, artificially reduces variance in that variable and the respective standard errors are thus deflated [13,16]. Furthermore, mean value imputation ignores the multivariate relationship between variables. A more advanced approach to handle missing data is conditional mean imputation [13]. In conditional mean imputation, a regression model is used to impute a single missing value conditional on the observed variables. A drawback of the conditional mean imputation method is that it amplifies the multivariate relationship between variables, and implies that after imputation, the missing value is known with certainty [13]. Finally, MI usually represents the most appropriate method to handle missing data as it respects that there is uncertainty with respect to the imputed value of a missing record [13,14,17,18]. In MI, multiple plausible values conditional on the observed data are generated for each missing record, resulting in multiple complete imputed datasets. The analysis is then performed for each of the imputed complete datasets and the results are thereafter pooled [13,14,17,18]. This allows the analysts to explicitly incorporate the uncertainty with respect to the true value of the missing record in the dataset. There are different statistical techniques to conduct MI such as MI by chained equations (MICE) or joint modeling (JM) [13,14,17,18]. From the 49 studies included, MICE to replace missing records has only been used by Stone et al. [44] and also in a reanalysis of the study by Sendi et al. [57,71]. Given the high prevalence of missing values of 38.8% due to absent information in the present study, this clearly shows that MI is underused in CV studies in dental medicine. It is certainly noteworthy that the present results are in line with other studies that evaluated how missing values were dealt with in medical research. For example, Vesin et al. reported that in clinical studies in intensive care, out of 44 published manuscripts, 45% reported missing values but only 2.5% used sophisticated methods to address this issue [15].

### 4.2. Zero Values

CV studies using WTP are a stated preference method where respondents directly express their valuation of a good or service, as opposed to other methods that assess the valuation of a commodity based on observed behavior using revealed preference methods such as discrete choice experiments [3,12]. CV methods were first used in environmental economics to value goods or services for which no market and, hence, price exists [72]. However, it has been recognized that CV is also a useful methodology for valuing a commodity in healthcare and its use in medicine, and more recently, also in dentistry which has substantially increased in recent years [1,2,71]. CV studies usually elicit the preference of respondents by asking them how much they are willing to pay for a good or service using a variety of different questioning methods such as open-ended questions, bidding games or payment cards [3,12,73]. WTP can also be interpreted as an alternative to other economic methods for valuing a health state such as the time-trade-off and standard gamble method. However, when using WTP, health benefits are directly expressed in monetary terms [10,11]. Nonetheless, there are also some potential biases associated with WTP elicitation methods such as strategic behavior, framing effects, response uncertainty and protest responses [19,20,74,75]. While respondents may express their protest by refusing to place a monetary value on a commodity, resulting in a missing value, some respondents may place a zero WTP on a service or good for non-economic reasons, such as their opinion that the government or insurance should cover the costs [19,20].

For example, insured individuals may value mandibular overdentures supported by two dental implants at zero WTP with the argument that they are not responsible for covering the costs (i.e., non-economic reason). This zero WTP valuation, however, does not reflect their true underlying preference and is hence termed as a protest zero. On the other hand, if non-insured individuals with otherwise no financial support value such a 2-implant mandibular overdenture with zero WTP, with the argument that the treatment is too expensive (i.e., economic reason), then the response is considered as a true zero and reflects the true underlying preference for the commodity. Protest zeros are a common problem in CV studies and may range from 50% up to 73% of all zero valuations and should be treated differently than true zeros [20]. In the present study, it was found that 57.1% of all WTP studies in dentistry reported zero values, and of those, only in 32.1% was a distinction made between true zeros and protest zeros. It has been suggested that in case of a zero valuation, the motivation for a zero valuation should be elicited in follow-up questions so that true zeros can be separated from protest zeros [19,20,21,75]. As some protest responses may also include a non-response in WTP elicitation, resulting in a missing value, it may be helpful to also evaluate the reason for a non-response using follow-up questions.

There is no common consensus on how zero values should be treated in analyzing the dataset [19,20]. However, methods to deal with protest responses can be separated into (i) those that deal with this issue in the data management phase (i.e., complete case analysis or imputation) and (ii) those that address zero values in the analysis phase (e.g., Heckman model, Tobit 2 model). The most commonly used approach is to remove protest zeros from the dataset [19,20]. However, this implies that those who protested are a representative subsample of those who did not, which is usually not true. Including protest zeros in the analysis leads to an underestimation of the mean WTP, and excluding protest zeros may lead to a distorted estimation of the mean WTP and reduced precision, i.e., a loss of statistical power. An alternative more advanced method is to treat protest responses as missing values and replace protest zeros with reasonable values using MI based on observed data such as socioeconomic variables and patient characteristics [19]. In a recent simulation study, it was shown that MI leads to less bias than addressing zero values in the analysis phase using alternative statistical models such as the Heckman model [19].

The Heckman selection model addresses non-response by adjusting for the probability of being a protester and, therefore, explicitly models that protesters may not be a representative subsample of the population analyzed. However, the Heckman model may be sensitive to other statistical issues such as deviations from the normality assumption, which is often encountered in practice [19]. In the present review, only one study used a Heckman model to address the problem of zero values in WTP elicitation. Other statistical models such as a Tobit 2 regression and the Double-Hurdle method have also been suggested to address non-responses [20,76]. However, these models do not make a distinction between protest zeros and true zeros per se; and their detailed discussion is beyond the scope of the present paper. The statistical methodology applied by the included studies is broad and also includes Tobit regression models, which account for the density of zero values (Table 1). Most of the included studies, however, used multivariate linear models (Table 1). Nevertheless, it should be noted that on a continuous linear scale, WTP values range from (true) zero to infinity and cannot be negative. The advantage of using MI for handling protest zeros in the data management phase is that it increases statistical power and does not predetermine the statistical model to be used when analyzing the data, so that any model can be used that best explains the data.

## 5. Conclusions

The present study shows that missing values and protest responses are common in CV studies in dental medicine, and that there is substantial uncertainty in addressing these issues. In cases of missing responses and zero WTP values, it is recommended to elicit additional information for the reason of such a response using follow-up questions. Zero WTP values should then be separated into true zeros due to economic reasons, and protest zeros based on non-economic reasoning. It is advisable to use multiple imputation to replace missing values due to missingness and protest zeros in the data management phase. This increases statistical power and does not predetermine the statistical model to be used in the analysis phase. The present study thus helps to clarify how missing values and protest zeros may be handled in future CV studies in dental medicine.

## Figures and Tables

**Table 1:** Contingent valuation willingness-to-pay studies included in review (sorted by date of publication).

Reference Year	Country	Intervention	Sample Size	Elicitation Method	Missing Values	Zero Values (True/Protest)	Distinction (True/Protest) Statistical Methodology
Dixon and Shackley, 1999 [21]	UK	Water fluoridation	93	Payment card	Yes (*n* = 30)	Yes (*n* = 12)	Yes Univariate analysis
Matthews et al., 1999 [22]	Canada	Periodontal treatment	42	Bidding game	Yes (*n* = 2)	Yes (*n* = 1)	Yes (true zero) ANOVA
Matthews et al., 2002 [23]	Canada	Anesthetic gel	293	Bidding game	No	Yes (13.3–27.3%)	No Univariate analysis
Birch et al., 2004 [24]	USA	Dentin regeneration	611	Open-ended	Yes (1.7–3.2%)	No	Multivariate linear regression
Halvorsen and Willumsen, 2004 [25]	Norway	Dental fear treatment	62	Open-ended	Yes (*n* = 8)	Yes (*n* = ?)	No Ordinary least squared corrected for heteroscedasticity
Pavlova et al., 2004 [26]	Bulgaria	Public healthcare services	990	Interval check-list and open-ended	No	Yes (*n* = ?)	No Tobit regression
Smith and Cunningham, 2004 [27]	UK	Orthognathic treatment	188	Payment card	No	Yes (>1)	No Multivariate linear regression
Tamaki et al., 2004 [28]	Japan	Dental check-up	5132	Payment options with bid amounts	Yes (*n* = 241)	No	ANOVA and multivariate logistic regression
van Steenberghe et al., 2004 [29]	Belgium	Anesthetic gel for hygiene treatment	157	Bidding game	Yes (*n* = 1)	Yes (*n* = 43)	Yes (f-up question) Tobit regression
Atchison et al., 2007 [30]	USA	Treatment of mandibular fracture	203	Continuous payment scale	No	No	Multivariate linear regression
Balevi and Shepperd, 2007 [31]	Canada	Management of an abscessed molar	40	Bidding game	No	Yes (<20%)	No (all included) ANOVA and decision-tree model
Oscarson et al., 2007 [32]	Sweden	Caries preventive dental care	82	Double-bounded dichotomo-us choice	No	No	Yes (but no zeros) Multivariate linear regression
Tianviwat et al., 2008 [33]	Thailand	Sealant and filling for children	205	Bidding game and open-ended	No	No	Multi-level linear regression
Tuominen, 2008 [34]	Finland	Check-up for 7-year-olds	156	Open-ended	No	No	Logistic/Linear regression
Esfandiari et al., 2009 [35]	Canada	Mandibular 2-implant overdenture	36	Open-ended and dichotomo-us choice	No	Yes (*n* = 4)	No Univariate analysis
Rosvall et al., 2009 [36]	USA	Orthodontic appliance	50	Payment scale	No	No	Multivariate linear mixed model (ANOVA)
Leung and McGrath, 2010 [37]	China	Single tooth replacement with implant	51	Bidding game	No	No	Multivariate linear regression
Al Garni et al., 2012 [38]	Saudi Arabia	Tooth replacement with implant	100	Bidding game	No	No	Binomial logistic regression
Ethier et al., 2012 [39]	Canada	Prevention of oral mucositis in children with cancer	142	Bidding game	No	Yes (*n* = ?)	No (all zeros as protesters and excluded) Interval regression
Feu et al., 2012 [40]	Brazil	Orthodontic appliance	252	Payment scale	No	No	Univariate analysis
Vermaire et al., 2012 [41]	Nether-lands	Parental investment in good oral health for children	290	Closed-ended question with payment ranges	No	Yes (20.7%)	No Standardized linear regression coefficients/bivariate and multivariate correlations
Widström and Seppälä, 2012 [42]	Finland	Replacing lost filling	987	Open-ended	Yes (*n* = 283)	No	Ordered logistic regression
Moshkelgosha and Golkari, 2013 [43]	Iran	Orthodontic treatment	192	Open-ended	Yes (*n* = 2)	No	ANOVA
Stone et al., 2013 [44]	UK	Personalized plaque control	39	Payment card	Yes (*n* = 3) MI	No	Multivariate linear regression
Augusti et al., 2014 [45]	Italy	Implant/crown versus FPD	107	Bidding game	No	No	Multivariate linear regression
Srivastava et al., 2014 [46]	Canada	Mandibular 2-implant overdenture	39	Payment scale with bid amounts	No	Yes (2.6–28.2%)	No Multivariate linear regression
Moshkelgosha et al., 2015 [47]	Iran	Orthodontic treatment	348	Payment scale	No	No	ANOVA
Re at al., 2015 [48]	Italy	Sonic/manual toothbrush/ oral hygiene treatment	40	Open-ended	No	No	Univariate analysis
Vernazza et al., 2015 [49]	UK/Germany	Coating for root caries prevention	112	Bidding card	Yes (*n* = 7)	Yes (*n* = ?)	No (all zeros set as true zeros, all missings set as protest) Linear/logistic/multinomial regression
Vernazza et al., 2015 [50]	UK	Molar RCT and Crown/gap/RPD/FPD/Implant	503	Payment card	No	Yes (*n* = 6)	Yes (f-up question, all true zeros) Logistic/multinomial/ Tobit regression/Heckman model
Atanasov et al., 2016 [51]	Bulgaria	RCT/crown versus extraction/implant	111	Open-ended	Yes (*n* = 4)	Yes (*n* = 1)	No Negative binomial regression
Farronato et al., 2016 [52]	Italy	Biomimetic orthodontic treatment	83	Bidding game	No	No	Multivariate linear regression model
Fatani and Al-Yousef, 2016 [53]	Saudi Arabia	Orthodontic treatment	171	Dichoto-mous and open-ended	Yes (*n* = 3)	No	Univariate analysis
McKenna et al., 2016 [54]	Ireland	RPD versus SDA versus Implant	55	Payment card	No	Yes (*n* = 4)	No Multivariate linear regression
Nair and Yee, 2016 [55]	Singapore	Extraction/filling/cleaning	83	Bidding game and open-ended	No	Yes (*n* = 16)	No Negative binomial regression
Re et al., 2017 [56]	Italy	RCT and crown vs. extraction, implant and crown	103	Bidding game	No	No	Univariate analysis
Sendi et al., 2017 [57]	Switzerland	Two interforaminal dental implants for denture retention	17	Bidding game	Yes (*n* = 1)	No	Univariate analysis
Berendsen et al., 2018 [58]	Nether-lands	Children’s oral health	630	Closed-ended question with payment ranges	Yes (*n* = 23)	Yes (*n* = 1.9%)	No Multivariate ordered logistic regression
Nyamuryekung’e et al., 2018 [59]	Tanzania	Tooth extraction and filling	1522	Open-ended	Yes (8.5–9.3%)	Yes (2–5%)	No Multivariate linear regression with Ln(1 + WTP) as response
Re et al., 2018 [60]	Italy	Computer-controlled anesthesia	50	Bidding game	No	Yes (*n* = 21)	No Univariate analysis
Vernazza et al., 2018 [61]	UK	Orthodontic treatment	401	Payment card	Yes (*n* = ?)	Yes (*n* = ?)	Yes (f-up questions) Tobit regression
Christell et al., 2019 [62]	Sweden	Osteoporosis risk assessment	144	Payment scale	Yes (*n* = 3)	Yes (*n* = 12)	Yes (f-up questions, included) ANOVA
Emami et al., 2019 [63]	Canada	Third midline implant for denture retention in addition to two interforaminal implants	17	Dichoto-mous and open-ended	No	Yes (53–70%)	No Univariate analysis
Walshaw et al., 2019 [64]	Brazil/UK	Fluoride varnish	200	Bidding card	No	No	Multivariate linear and Tobit regression
Harris et al., 2020 [65]	UK	Risk-information during check-up	412	Payment card and double bounded dichoto-mous choice	Yes (*n* = 21)	Yes (*n* = ?)	Yes (f-up questions, protesters excluded) MANCOVA and Tobit regression
Sever et al., 2020 [66]	Croatia	Dental care delivery	242	Payment card	Yes (*n* = 22)	Yes (included in missings)	No Interval regression model
Srivastava et al., 2020 [67]	Canada	Mandibular 2-implant overdentures	317	Triple-bounded discrete choice with open-ended termination	No	Yes (1.3–7.3%)	No (zeros included) Lognormal regression models
Saadatfar and Jadidfard, 2021 [68]	Iran	Fissure sealant and filling in molars	290	Payment card and open-ended	No	Yes (*n* = 3)	Yes Multivariate linear regression
Akuagwuag-wu et al., [69]	UK	Scaling and polish	1405	Payment scale	No	Yes (*n* = 117)	Yes (protest zeros excluded) Interval regression

Abbreviations: FPD denotes fixed partial denture, RPD denotes removable partial denture, SDA denotes shortened dental arch, RCT denotes root canal treatment, MI denotes multiple imputation, f-up denotes follow-up.

## Data Availability

Not applicable.

## Supplementary Materials

The following are available online at https://www.mdpi.com/article/10.3390/ijerph18147219/s1.
