# Supplementary material for: Prevalence of Missing Values and Protest Zeros in Contingent Valuation in Dental Medicine
Source: Int J Environ Res Public Health. 2021 Jul 6;18(14):7219. doi: 10.3390/ijerph18147219 (PMC8307611; doi:10.3390/ijerph18147219)

Search strategy

Search was conducted on June 8, 2021

PubMed search strategy

#	search	results
1	“Willingness to pay”	6510
2	WTP	2147
3	“Willingness to accept”	797
4	WTA	570
5	“Contingent valuation”	873
6	“Conjoint analysis”	876
7	“Cost benefit analysis”	86892
8	“Discrete choice experiment”	1730
9	“Monetary value”	694
10*	1 OR 2 OR 3 OR 4 OR 5 OR 6 OR 7 OR 8 OR 9	94545
11	Dentistry [MeSh]	413982
12	“dent”	313226
13*	11 OR 12	567501
14*	10 AND 13	1357

*Preceding search criteria are combined

Web of Science search strategy

#	search	results
1	“Willingness to pay”	20645
2	WTP	6513
3	“Willingness to accept”	1803
4	WTA	3200
5	“Contingent valuation”	6834
6	“Conjoint analysis”	4549
7	“Cost benefit analysis”	15106
8	“Discrete choice experiment”	3013
9	“Monetary value”	2377
10*	1 OR 2 OR 3 OR 4 OR 5 OR 6 OR 7 OR 8 OR 9	50208
11	Topic (“dent*)	334251
12*	10 AND 11	193

*Preceding search criteria are combined

Cochrane Library search strategy

#	search	results
1	“Willingness to pay”	1505
2	WTP	311
3	“Willingness to accept”	109
4	WTA	62
5	“Contingent valuation”	61
6	“Conjoint analysis”	44
7	“Cost benefit analysis”	9866
8	“Discrete choice experiment”	222
9	“Monetary value”	83
10*	1 OR 2 OR 3 OR 4 OR 5 OR 6 OR 7 OR 8 OR 9	11248
11	Dentistry [MeSh]	17989
12	“dent*”	1299
13*	11 OR 12	18940
14*	10 AND 13	127

* Preceding search criteria are combined

PROSPERO search strategy

#	search	results
1	“Willingness to pay”	141
2	WTP	40
3	“Willingness to accept”	12
4	WTA	1
5	“Contingent valuation”	19
6	“Conjoint analysis”	36
7	“Cost benefit analysis”	319
8	“Discrete choice experiment”	35
9	“Monetary value”	27
10*	1 OR 2 OR 3 OR 4 OR 5 OR 6 OR 7 OR 8 OR 9	500
11	Dentistry	4020
12	“dent*”	9245
13*	11 OR 12	9245
14*	10 AND 13	28

* Preceding search criteria are combined

Figure S1. PRISMA (Preferred Reporting Items for Systematic Reviews and Meta-Analyses) flow diagram.

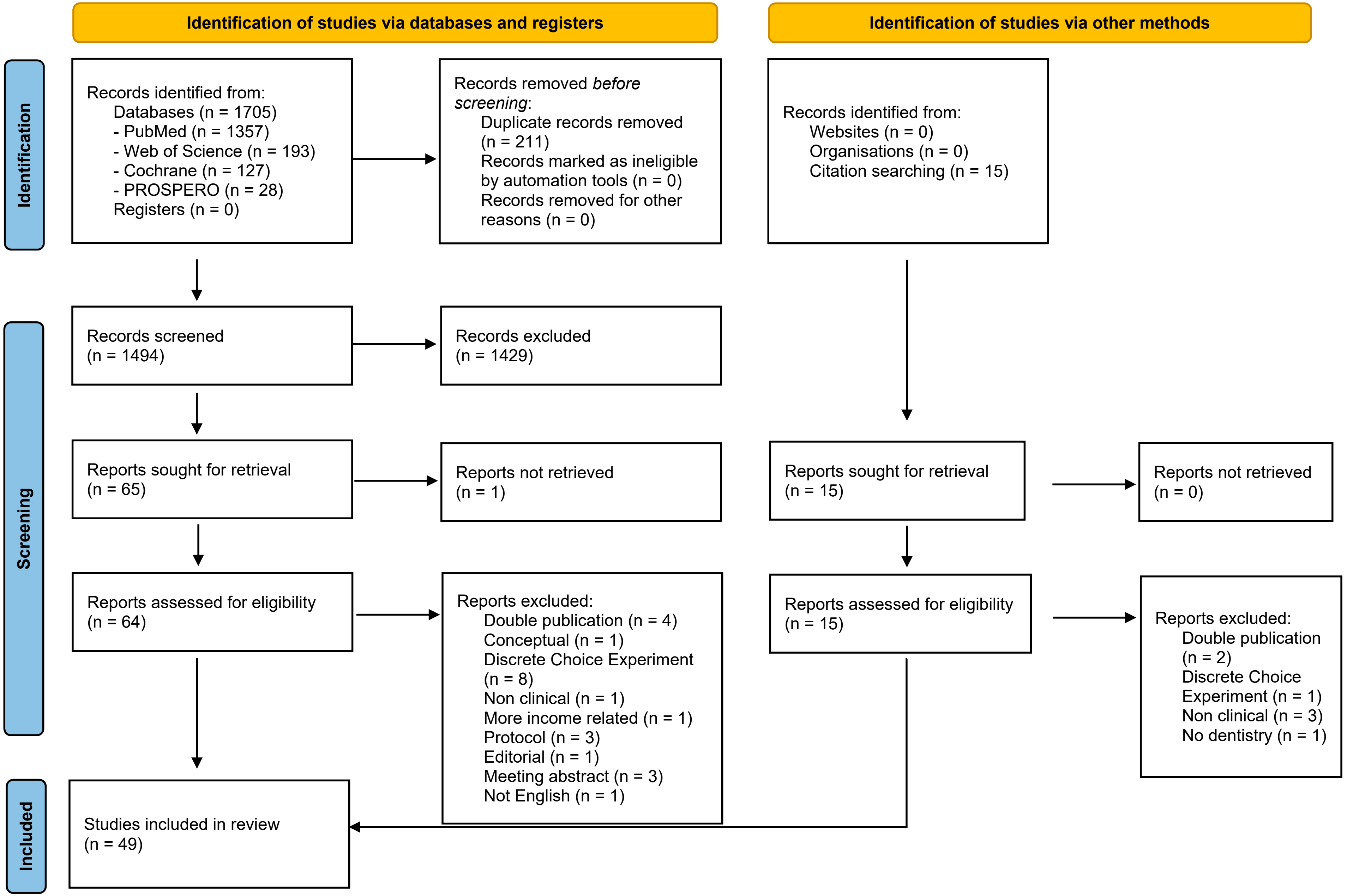

Supplement: Supplementary file 1 [file ijerph-18-07219-s001.zip › ijerph-1239827-supplementary.pdf]
